# Quercetin and Ferulic Acid Elicit Estrogenic Activities In Vivo and In Silico

**DOI:** 10.3390/molecules28135112

**Published:** 2023-06-29

**Authors:** Meryem Slighoua, Fatima Ez-Zahra Amrati, Mohamed Chebaibi, Ismail Mahdi, Omkulthom Al Kamaly, Khadija El Ouahdani, Aziz Drioiche, Asmaa Saleh, Dalila Bousta

**Affiliations:** 1 Laboratory of Biotechnology, Environment, Agro-Food, and Health (LBEAS), Faculty of Sciences, University 7 Sidi-Mohamed-Ben-Abdellah (USMBA), Fez 30050, Moroccoelouahdanikhadija@gmail.com (K.E.O.);; 2 Biomedical and Translational Research Laboratory, Faculty of Medicine and Pharmacy of Fez, Sidi Mohamed Ben Abdellah University, Fez 30000, Morocco; mohamed.chebaibi@yahoo.fr; 3 AgroBioSciences Research Division, College for Sustainable Agriculture and Environmental Science, Mohammed VI Polytechnic University, Lot 660-Hay Moulay Rachid, Ben Guerir 43150, Morocco; ismail.mahdi@um6p.ma; 4 Department of Pharmaceutical Sciences, College of Pharmacy, Princess Nourah Bint Abdulrahman University, P.O. Box 84428, Riyadh 11671, Saudi Arabia; omalkmali@pnu.edu.sa; 5 Laboratory of Innovative Materials and Biotechnology of Natural Resources, Faculty of Sciences, Moulay 19 Ismail University, Meknes 50070, Morocco; drioichelabo@gmail.com

**Keywords:** quercetin, ferulic acid, sub-acute toxicity, female infertility, estrogenic activity, docking study

## Abstract

In this study, we examined the sub-acute toxicity of quercetin and ferulic acid and evaluated their effects on protein, cholesterol, and estrogen levels in vivo. Six groups of female Wistar rats were fed by gavage. The first and second groups represent the positive (Clomiphene citrate 10 mg/kg) and negative (NaCl 0.9%) control groups, while the other groups received quercetin and ferulic acid at doses of 5 and 10 mg/kg/day for 28 days. The sub-acute toxicity was monitored by examining the weights, biochemical parameters (AST, ALT, ALP, urea, and CREA), and histological changes in the kidneys and liver of the treated animals. Furthermore, the in vivo estrogenic effects were studied in terms of the serum and ovarian cholesterol levels, serum estradiol, and uterine proteins. Finally, Docking studies were conducted to evaluate the binding affinity of quercetin and ferulic acid for alpha and beta estrogen receptors. Results showed that both compounds were devoid of any signs of nephrotoxicity or hepatotoxicity. Additionally, quercetin and ferulic acid caused significant estrogenic effects evidenced by an increase of 8.7 to 22.48% in serum estradiol, though to a lesser amount than in the reference drug-treated group (64.21%). Moreover, the two compounds decreased the serum cholesterol levels (12.26–32.75%) as well as the ovarian cholesterol level (11.9% to 41.50%) compared to the negative control. The molecular docking in estrogen alpha and estrogen beta active sites showed high affinity of quercetin (−10.444 kcal/mol for estrogen alpha and −10.662 kcal/mol for estrogen beta) and ferulic acid (−6.377 kcal/mol for estrogen alpha and −6.3 kcal/mol for estrogen beta) to these receptors. This study provides promising insights into the potential use of quercetin as a therapeutic agent for the management of female fertility issues.

## 1. Introduction

Infertility is diagnosed when a couple is unable to conceive within six months for women over 35 and one year for women under 35. The inability to carry a pregnancy to term and deliver a living child is another definition of infertility [1]. In the case of primary infertility, the couple could never conceive. Difficulties arise with secondary infertility related to post-conception and conception (either full term or abortion). Secondary infertility is not present if partners change within a year. This is related to the male partner’s special possibility of infertility [2]. Infertility can be caused by various factors affecting either the man, the woman, or both. Medical conditions such as uterine fibroids, polycystic ovarian syndrome, endometriosis, pelvic inflammatory disease, premature ovarian failure, and environmental factors can all contribute to this dysfunction [3]. Poor semen quality is the main cause of male infertility [4], while ovulation issues, tubal obstruction, age-related factors, uterine issues, prior tubal ligation, and hormone imbalances can lead to female infertility [5]. The mechanisms by which multivariate factors affect female fertility are still not well understood. Some studies have proposed that the high frequency of this disease is likely to increase as postponement of childbearing increases, mainly in developed parts of the world [6].

A variety of medical treatments are available for infertility, encompassing the utilization of fertility medications to induce “superovulation,” which involves the development and release of multiple eggs during each ovulatory cycle, along with intrauterine insemination and advanced assisted reproductive technologies [7]. Nevertheless, these medical interventions pose significant financial burdens for individuals in developing nations and do not guarantee consistent efficacy or safety.

While there are many pharmaceuticals available to treat female infertility, there is increasing interest in alternative and complementary therapies, such as plant-based remedies. Herbal medicines have been used for centuries to treat reproductive problems, and there is evidence suggesting that certain plants can effectively treat infertility in women [8,9]. This field of study is known as herbal gynecology, and it has gained popularity in recent years. Many plants have been identified as having potential therapeutic effects for female infertility, such as *Senecio biafrae*, *Petroselinum sativum*, and *Lavandula officinalis* [10,11,12]. Indeed, it has long been known that a wide range of exogenous substances such as xenoestrogens, phytoestrogens, and steroidal estrogens can mimic the activities of endogenous estrogen to different degrees [13]. Phytoestrogens, such as resveratrol and catechin, are found in various foods and have been shown to have antioxidative properties [14] and beneficial effects on neurological disorders [15]. These compounds have the ability to modulate the effects of estrogens in the body and are therefore often referred to as “dietary estrogens”. Phytoestrogens are found in a wide variety of foods, including soybeans, flaxseed, lentils, chickpeas, and many fruits and vegetables. In recent years, they have become increasingly popular due to their potential health benefits, specifically in addressing menopausal symptoms and promoting cardiovascular health [16,17].

In our previous studies, we investigated the estrogenic effect of *Petroselinum sativum* [12] and *Lavandula officinalis* [11] extracts and profiled their phytochemical constituents using HPLC. Interestingly, these studies demonstrated that ferulic acid and quercetin had the highest concentration among the identified molecules. According to these results, we thought to evaluate the effect of these two phytoconstituents of the female reproductive system. The main goal was to find a preliminary alternative based on medicinal plants rich in quercetin or ferulic acid instead of synthetic chemicals having undesirable effects both in the short or long term, such as psychological side-effects of clomiphene citrate and human menopausal gonadotrophin.

Numerous studies have demonstrated the implications of phenolic compounds from medicinal plants on the regulation of reproductive functions [11,18]. The flavonol subclass of flavonoid chemicals includes quercetin, which has a variety of potential positive impacts on human health [19]. Numerous investigations have revealed that quercetin exhibits a diverse array of biological effects, encompassing antioxidant, anti-inflammatory, and anti-aging properties. Consequently, quercetin was reported to significantly reduce the risk of developing cancer, diabetes, obesity, and other illnesses [20]. Ferulic acid, one of the most frequent phenolics, is found in a variety of plants, especially cereals, fruits, and vegetables [21]. It is closely related to cinnamic acid and results from the metabolism of phenylalanine and tyrosine by the Shikimate pathway. Facilitating the release of ferulic acid from the cell wall poses a challenge due to its predominant presence in the form of ester cross-links with polysaccharides. These polysaccharides, such as arabinoglycans in grasses, pectin in spinach, sugar beet, and xyloglycans in bamboo, contribute to the difficulty in releasing ferulic acid [22].

In this study, we explored the estrogenic potential of quercetin and ferulic acid in female Wistar rats by assessing their effects on serum and ovarian cholesterol levels, serum estradiol, and uterine proteins. In addition, an in silico study was performed to assess the affinity of both quercetin and ferulic acid for estrogen receptors.

## 2. Results

### 2.1. Sub-Acute Toxicity of Ferulic Acid and Quercetin

#### 2.1.1. Monitoring Animal Body Weight and Signs of Toxicity

**Figure 1:**
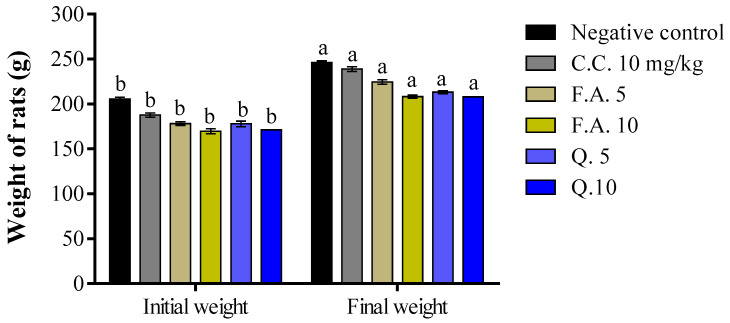
The impact of quercetin and ferulic acid on weight gain was assessed before and after a 28-day treatment period. The superscript letters indicate significant differences at a *p*-value of less than 0.05 between the treatments. The values provided represent the mean ± standard error of the mean. The negative control group was administered NaCl 0.9%, while the C.C. group received clomiphene citrate, the F.A. group received ferulic acid, and the Q. group received quercetin.

Throughout the 28-day treatment period, all animals exhibited significant weight gain (Figure 1). However, no signs of toxicity were observed such as changes in behavioral responses (locomotion, aggression), spontaneous activity (response to tail nipping and noise), stool appearance, tremors, bristling, and mortality. Indeed, body weight gain was induced by all doses of both quercetin and ferulic acid, as well as by the two control treatments.

#### 2.1.2. Evaluation of Biochemical Parameters

**Figure 2:**
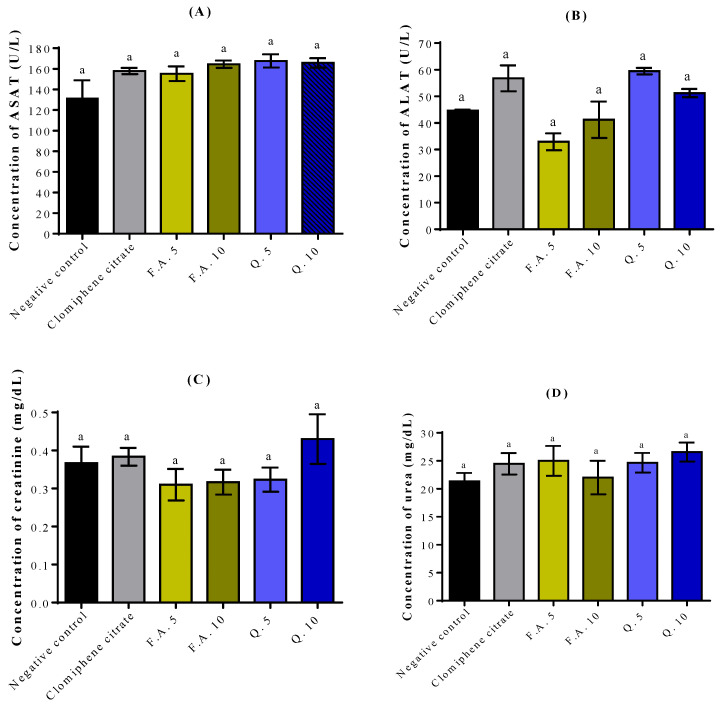
Effect of quercetin and ferulic acid on liver (**A**,**B**) and kidney (**C**,**D**) parameters. The letters in superscript indicate the significant difference at *p* < 0.05 between treatments. Negative control (NaCl 0.9%), F.A. (Ferulic Acid), Q. (Quercetin).

To assess the effect of the different treatments on the renal and hepatic functions, we analyzed the relevant serum parameters, mainly ALAT, ASAT, urea, and creatinine. Our findings demonstrated that the administration of quercetin and ferulic acid at doses of 5 and 10 mg/kg did not result in any adverse effects on hepatic parameters, as evidenced by ALAT (Figure 2A) and ASAT (Figure 2B), nor on renal parameters, including UREA (Figure 2C) and CREA (Figure 2D).

#### 2.1.3. Organ Histopathology

**Figure 3:**
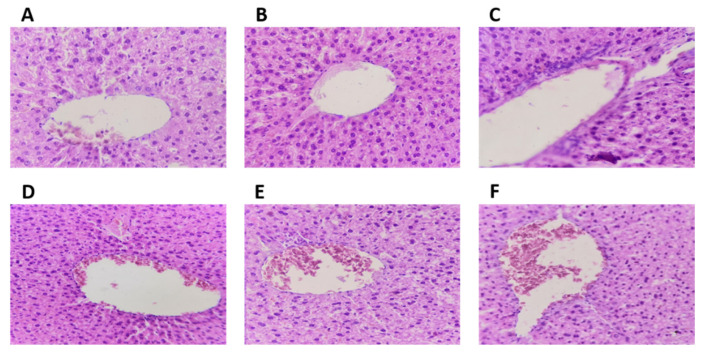
Liver histopathological sections were stained with hematoxylin–eosin–safran at a magnification of ×40: (**A**) represent the negative control (NaCl 0.9%); (**B**) represent the reference group treated with clomiphene citrate (10 mg/kg); (**C**,**D**) represent ferulic acid groups (5 and 10 mg/kg, respectively); (**E**,**F**) represent quercetin groups (5 and 10 mg/kg, respectively).

The examination of tissue samples treated with quercetin and ferulic acid under a microscope revealed no histological abnormalities in the liver and kidneys. Specifically, there were no observable changes or histopathological abnormalities in the structures of the central vein, hepatocytes, and hepatic sinusoids, indicating the absence of liver toxicity (Figure 3).

**Figure 4:**
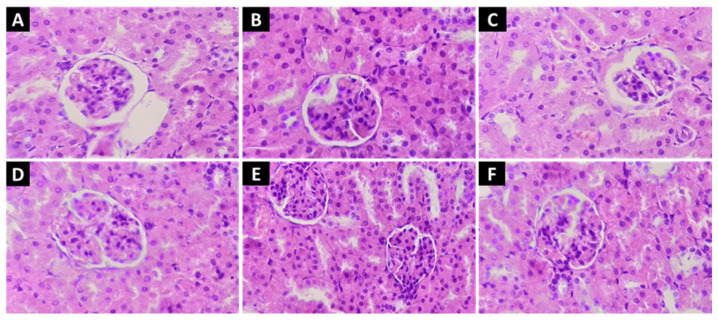
Kidney histopathological sections were stained with hematoxylin–eosin–safran at a magnification of ×40: (**A**) represents the negative control (NaCl 0.9%); (**B**) represents reference group treated with clomiphene citrate (10 mg/kg); (**C**,**D**) represent ferulic acid groups (5 and 10 mg/kg, respectively); (**E**,**F**) represent quercetin groups (5 and 10 mg/kg, respectively).

Likewise, the examination of histological sections of the kidneys revealed that none of the administered doses had an impact on renal structures, including the glomerulus, proximal convoluted tubules, and distal convoluted tubules (Figure 4).

### 2.2. Ovarian Stimulation Test

#### 2.2.1. Weight of Ovaries and Uterus

**Figure 5:**
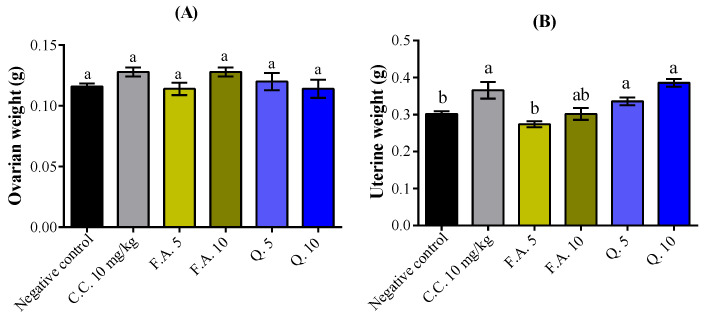
Impact of quercetin and ferulic acid on (**A**) ovarian and (**B**) uterine weights was assessed in comparison to the negative control (NaCl 0.9%) and positive control (clomiphene citrate (C.C)). The letters in superscript indicate the significant difference at *p* < 0.05 between treatments. The data represent the mean ± s.e.m. (*n* = 5). F.A. (ferulic acid), Q. (quercetin).

Following 28 days of treatment, all treatments induced no significant difference in the weight of ovaries (Figure 5A). However, uterine weights showed significant increases by quercetin of 11.25% and 27.81% at the doses of 5 and 10 mg/kg, respectively (*p* < 0.01), with comparable performance to the standard drug, clomiphene citrate (Figure 5B).

#### 2.2.2. Ovarian and Serum Cholesterol Assay

**Figure 6:**
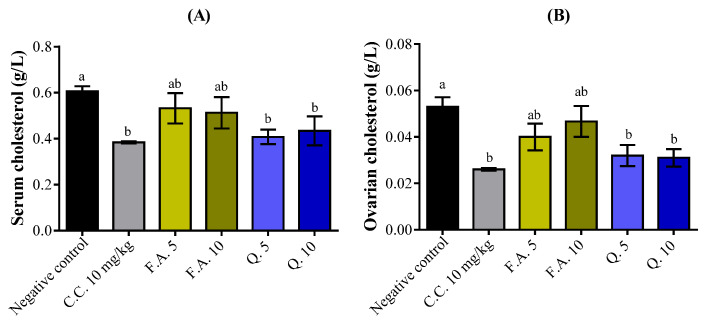
The impact of quercetin and ferulic acid on the levels of (**A**) serum and (**B**) ovarian cholesterol was assessed. The superscript letters indicate significant differences at a *p*-value of less than 0.05 among the treatments. The presented data represent the mean ± standard error of the mean (*n* = 5). Negative control (NaCl 0.9%), C.C. (clomiphene citrate), F.A. (ferulic acid), Q. (quercetin).

Figure 6 depicts the serum and ovarian cholesterol biochemical alterations following 28 days of oral administration of ferulic acid and quercetin to mature rats. Serum cholesterol levels decreased significantly at the doses of 5 and 10 mg/kg of quercetin by 32.75% and 28.46%, respectively (*p* < 0.001) (Figure 6A). Similarly, ovarian cholesterol levels also decreased significantly at the same doses of quercetin by 32.62% and 41.50%, respectively (*p* < 0.001), compared to the negative control group (Figure 6B). In contrast, no significant changes were observed using ferulic acid as compared to the negative control group.

#### 2.2.3. Uterine Protein and Serum Estradiol Assay

**Figure 7:**
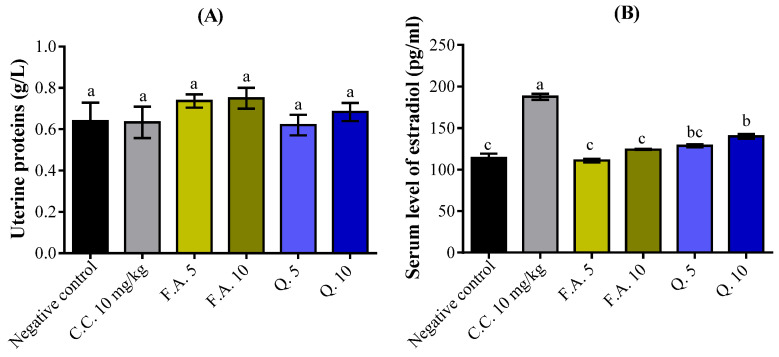
The impact of quercetin and ferulic acid on the levels of uterine proteins (**A**) and serum estradiol (**B**) were examined in comparison to the negative control (0.9% NaCl) and positive control (clomiphene citrate (C.C)). The letters in superscript indicate a significant difference at *p* < 0.05 between treatments. The values represent the mean ± s.e.m. F.A. (ferulic acid), Q. (quercetin).

After 28 days of treatment, no significant difference was noticed in the uterine protein levels (Figure 7A). Moreover, we observed a significant increase in serum estradiol levels following the administration of quercetin at the dose of 10 mg/kg by 22.48% compared to the negative control group (Figure 7B). Ferulic acid at both concentrations did not affect the serum estradiol.

#### 2.2.4. Histopathology of the Ovaries

**Figure 8:**
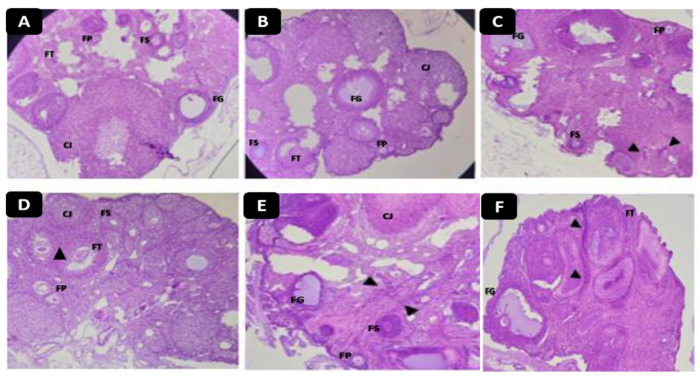
Histopathological sections of rat ovaries: (**A**) represents the negative control (NaCl 0.9%); (**B**) represents the reference group treated with clomiphene citrate (10 mg/kg); (**C**,**D**) represent ferulic acid groups (5 and 10 mg/kg, respectively); (**E**,**F**) represent quercetin groups (5 and 10 mg/kg, respectively). Abbreviations: FP: Primary Follicle; FS: Secondary Follicle; TF: Tertiary Follicle; FG: Graf’s follicle; CJ: Yellow Body; arrowheads: dilated blood vessels.

The results of the histological sections of the ovaries (Figure 8) treated with quercetin and ferulic acid showed no abnormality in their structures after observation of the different follicular stages (primary follicle, secondary follicle, tertiary follicle, follicle de Graff, corpus lutea). The triangles in the figures show the presence of dilated blood vessels filled with erythrocytes, which are characteristic symptoms of estrogenic activity. They were observed in almost all ovarian structures (Figure 8).

### 2.3. Docking Analysis of Quercetin and Ferulic Acid on Estrogen Alpha and Estrogen Beta Active Sites

The glide gscore, glide emodel, and glide energy for quercetin in estrogen alpha’s active site were −10.444, −82.345, and −54.171 kcal/mol, respectively. Moreover, quercetin showed in the estrogen beta receptor active site a glide gscore, glide model, and glide energy of −10.662, −74.309, and −51.529 kcal/mol (Table 1).

In the active site of estrogen alpha, the ferulic acid showed a glide gscore, glide emodel, and glide energy of −6.377, −39.698, and −27.718 kcal/mol. Ferulic acid showed in the active site of estrogen beta a glide gscore, glide model, and glide energy of −6.3, −38.943, and −27.308 kcal/mol (Table 1).

**Figure 9:**
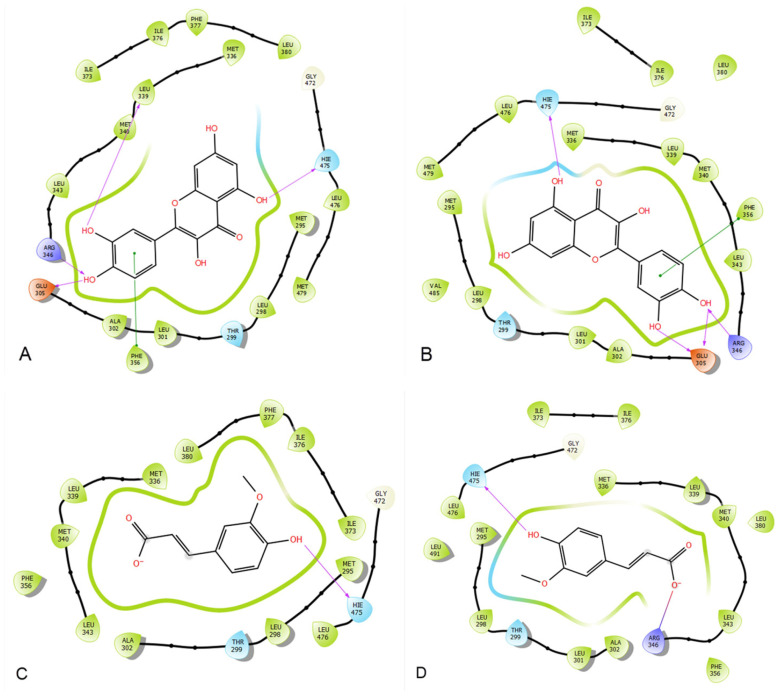
The two-dimensional viewer of ligands in active sites. (**A**) represents quercetin interactions in the active sites of estrogen alpha. (**B**) represents quercetin interactions in the active sites of estrogen beta receptors. (**C**) represents ferulic acid interactions in the active sites of estrogen alpha. (**D**) represents ferulic acid interactions in the active sites of estrogen beta receptors.

**Figure 10:**
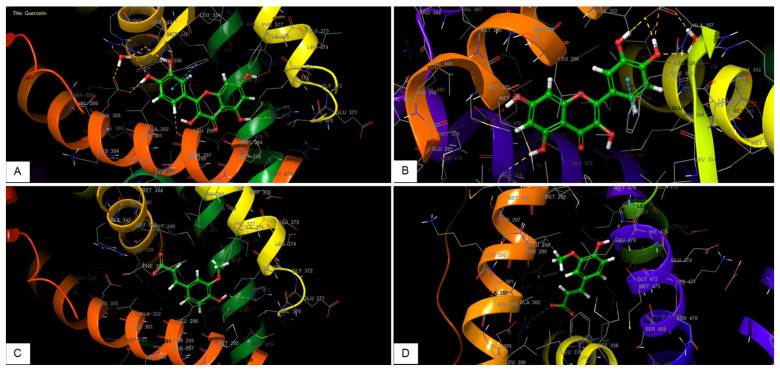
The three-dimensional viewer of ligands in active sites. (**A**) represents quercetin interactions in the active sites of estrogen alpha. (**B**) represents quercetin interactions in the active sites of estrogen beta receptors. (**C**) represents ferulic acid interactions in the active sites of estrogen alpha. (**D**) represents ferulic acid interactions in the active sites of estrogen beta receptors.

When quercetin was docked into the active site of estrogen alpha, it was observed that it formed four hydrogen bonds with GLU 305, ARG 346, LEU 339, and HIE 475 residues, along with a Pi–Pi stacking interaction with residue PHE 356. Furthermore, the docking of quercetin in the active site of Beta estrogen showed the formation of the same bonds with the same residues (Figure 9 and Figure 10).

In the active site of estrogen alpha, the docking of ferulic acid presented the formation of a single hydrogen bond with the residue HIE 475, whereas the docking of this molecule in the active site of estrogen beta showed the formation of one hydrogen bond with residue HIE 475 and one salt bridge with residue AGR 346 (Figure 9 and Figure 10).

## 3. Discussion

In this study, we examined the sub-acute toxicity of quercetin and ferulic acid and evaluated their effects on protein, cholesterol, and estrogen levels in vivo. We provided evidence that both quercetin and ferulic acid are devoid of any toxicity towards the two key organs of the metabolic processes and detoxification, namely, the liver and kidneys [23]. In addition, a significant increase in uterine weight was observed in mature female rats after 28 days of treatment with high doses of quercetin and ferulic acid. The reason behind this could be attributed to the stimulation of cell proliferation caused by estrogens and their precursors [9]. Nevertheless, concurrent research has brought attention to potential detrimental health consequences associated with quercetin and ferulic acid. Quercetin demonstrates genotoxicity in different in vitro systems, even in the absence of metabolic activation [24,25]. The mutagenic activity of quercetin is believed to be influenced by its recognized pro-oxidant activity [26]. Furthermore, subcutaneous implantation of estradiol implants in a rodent exposed to quercetin long-term enhanced carcinogenesis [27].

The proliferation of human breast cancer MCF7 cells is stimulated by ferulic acid in a concentration- and estrogen-receptor-dependent manner. This effect is associated with the upregulation of HER2 and estrogen receptor α expression [28].

The fact that cholesterol acts as the main precursor for hormones and steroids is widely recognized [11,29]. Here, we noticed a considerable decrease in ovarian and serum cholesterol levels in animals subjected to quercetin treatment. In fact, a drop in cholesterol levels is indicative of its critical involvement in steroidogenesis and in promoting the proliferation of ovarian cells. A notable decrease in cholesterol level was used to illustrate similar results using the doses of 10–50 mg/kg of quercetin [30]. Some studies have suggested that phytoestrogens can have a protective effect on the ovaries by reducing the risk of certain types of ovarian cancer [31]. This effect may be due to the ability of phytoestrogens to modulate the signaling pathways involved in cell growth and differentiation, as well as through their anti-inflammatory and antioxidant properties [32]. For instance, genistein, an isoflavone isolated from soybean, was shown to promote the production of ovarian progesterone, estradiol, and cAMP in animals, as well as the maturation of oocytes and the development of preimplantation zygotes [33].

Estradiol is a key hormone in female reproductive function. In our study, we observed a significant rise in estradiol in the quercetin group at the dose 10 mg/kg compared to the negative control group. The presence of estradiol-inducing secondary metabolites in plants, such as lignans, daidzein, and genistein, was proposed as a potential explanation for the observed similar induction [10,11,12]. The effect of flavonoids on serum estradiol levels may depend on the specific type and the amount of flavonoid [34]. For example, some studies have found that high doses of genistein, a flavonoid found in soybeans, may have an estrogenic effect and increase serum estradiol levels [35]. However, other studies have found that low doses of genistein may have an anti-estrogenic effect and decrease serum estradiol levels [36].

Phytoestrogens are a group of naturally occurring compounds found in plants that have a similar structure to the estrogen hormone [37]. The mechanism of action of phytoestrogens involves binding to estrogen receptors in the body, which can activate or inhibit various biological process [38]. They can also affect the synthesis, metabolism, and transport of hormones [39]. Phytoestrogens can act as agonists or antagonists of estrogen receptors, depending on the tissue type and the specific receptor subtype involved [40]. They can also exhibit selective estrogen receptor modulator (SERM) activity. This means that they can have different effects on different tissues and organs in the body [41].

Quercetin and ferulic acid showed a vasodilating effect of estrogen, which is partly linked to its ability to improve the bioavailability of nitric oxide (NO) [42,43], which is a powerful regulator of platelet aggregation, blood pressure, vascular smooth muscle mitogenesis, and leukocyte adhesion [44].

In addition to their capability to bind to estrogen receptors, phytoestrogens exert other biological effects that are independent of these receptors. These effects include the activation of serotoninergic receptors, IGF-1 receptors, and the ability to bind free radicals. They can also induce DNA methylation and affect various intracellular regulators such as tyrosine kinase, cAMP/protein kinase A, cGMP/NO, phosphatidylinositol-3 kinase/Akt, and MAP kinases (ERK1,2, p38). Moreover, they have the capacity to impact transcription factors like NF-kappaB and DNA topoisomerase activities, histone modification, RNA expression, and various intracellular regulators associated with cell cycle and apoptosis [45].

Estrogen receptors play a crucial role in the regulation of female infertility. Estrogen is a hormone that is essential for the development and function of the female reproductive system. Estrogen receptors are proteins that are located in various tissues of the body, including the uterus, ovaries, and hypothalamus [46]. In the ovaries, estrogen receptors are involved in the regulation of follicle development and ovulation. Estrogen helps to stimulate the growth and maturation of ovarian follicles, which contain developing eggs. Once the follicle is mature, estrogen levels rise rapidly, which triggers ovulation and the release of the egg from the ovary [47]. In the uterus, estrogen receptors help to prepare the lining of the uterus for the implantation of a fertilized egg. Estrogen promotes the growth and thickening of the uterine lining, known as the endometrium, which provides a nourishing environment for a developing embryo [48]. In the hypothalamus, estrogen receptors are involved in the regulation of the menstrual cycle. Gonadotropin-releasing hormone (GnRH) is produced by the hypothalamus, and it triggers the release of follicle-stimulating hormone (FSH) and luteinizing hormone (LH) from the pituitary gland. Estrogen feedback to the hypothalamus helps to regulate the levels of FSH and LH, which are essential for follicle development and ovulation [49].

In this work, an in silico investigation was conducted to examine the affinity of quercetin towards estrogen alpha and beta receptors. Interestingly, we found that quercetin exhibited remarkable binding activity of −10.444 and −10.662 kcal/mol, respectively. Likewise, ferulic acid, a compound that has been reported for its role in female infertility [50,51], also showed a strong affinity for these receptors, with a glidescore of −6.377 and −6.3 kcal/mol. This indicates that the estrogen-like impact of quercetin is probably caused by its interaction with estrogen receptors, which then trigger transcriptional processes and/or signaling events. These processes could ultimately regulate gene expression related to the development and function of the reproductive system. Nevertheless, further study is needed to monitor the epigenetic-like effect of quercetin.

The ability of quercetin and ferulic acid to induce apoptosis in different cancer cell lines has been extensively studied [52,53,54]. Quercetin and ferulic acid exert their apoptotic effect through multiple mechanisms. One of the key pathways involves the activation of the intrinsic or mitochondrial pathway. They trigger the release of cytochrome c from the mitochondria, which subsequently activates caspase enzymes, leading to cell death. Moreover, quercetin and ferulic acid have been shown to inhibit anti-apoptotic proteins, such as Bcl-2 and Bcl-xL, thereby promoting apoptosis [55,56,57].

This study shed light on the promising insights regarding the potential use of quercetin as a therapeutic agent for the management of female fertility issues through its interaction with estrogen receptors. Recent studies have unveiled its ability to modulate estrogen receptor signaling pathways, suggesting its potential to regulate estrogen levels and improve reproductive outcomes. By targeting estrogen receptors, quercetin may play a crucial role in maintaining hormonal balance, promoting follicular development, and enhancing ovulation. Furthermore, its anti-inflammatory effects could alleviate reproductive disorders associated with inflammation. While further research is necessary to fully elucidate the mechanisms involved, these promising findings pave the way for potential therapeutic applications of quercetin in the realm of female fertility, offering hope for improved management and treatment of reproductive challenges.

## 4. Materials and Methods

### 4.1. Preparation of Molecules

Ferulic acid and quercetin were obtained from Sigma Aldrich (St. Louis, MO, USA).

### 4.2. Animal Handling and Housing

Wistar rats with an average weight of 192 ± 18 g were used for the in vivo experiments. Animals were bred in the animal house of faculty of science in Fez, Morocco. The animals were raised in the animal facility at the Faculty of Science situated in Fez, Morocco, where the temperature was maintained at 22 ± 2 °C and a D/N cycle of 12 h/12 h was used. Animals were provided with unrestricted access to both food and water. The protocol for animal care and use has been submitted to the institutional ethics committee in accordance with the European Community Directive EEC/86/EEC [58].

### 4.3. Study Design

Quercetin and ferulic acid were dissolved in distilled water and orally administered to rats [59]. Treatments were considered based on the body weight of the animals, and the experiment was conducted over 28 days [60]. The negative control group received physiological solution (NaCl 0.9%). The second group was the positive control group, in which animals were treated with clomiphene citrate (10 mg/kg). In the third and fourth groups, ferulic acid was administered at doses of 5 and 10 mg/kg, respectively. Similarly, the fifth and sixth groups were given quercetin at doses of 5 and 10 mg/kg, respectively.

### 4.4. Sub-Acute Toxicity Assessment

To assess the sub-acute toxicity of quercetin and ferulic acid, the guideline no. 407 was followed [61]. The weight of the animals and their signs of toxicity, such as changes in behavioral responses (locomotion, aggression), spontaneous activity (response to tail nipping and noise), stool appearance, tremors, bristling, and mortality, were examined daily. At the end of the test, the rats were anesthetized with Pentobarbital and dissected. Blood was collected through cardiac puncture for biochemical parameter analysis, specifically aspartate aminotransferase (AST) and alanine transaminase (ALT) assays using EDTA tubes, as well as creatinine (CREA) and urea. The liver and kidney tissues were preserved in formalin solution (10%) until histopathological examination.

### 4.5. Ovarian Stimulation Test

To evaluate the ovarian stimulation effect of quercetin and ferulic acid, the animals were dissected after 28 days of treatment. Initially, the uterus and ovaries were weighed and pulverized in a NaCl (0.9%) solution. After centrifugation, the resulting supernatants were collected to evaluate the levels of ovarian cholesterol and uterine protein. This assessment was conducted using the enzymatic colorimetric technique with peroxidase (POD) for cholesterol and the biuret method for protein. [62]. The blood was collected and centrifuged for 10 min to recover the serum which was used to determine the level of cholesterol and estradiol using the enzyme-linked fluorescence assay (ELFA) [12,63].

#### Histopathology of the Ovaries

The ovaries were preserved in a 10% formalin solution. Then, histological examination was performed using standard techniques of microscopic observation via staining with hematoxylin and eosin [64].

### 4.6. In silico Analysis

*Estrogens are the key hormones involved in the regulation of fertility.* These two hormones are necessary for the cellular responses mediated by estrogen receptor α and β [65]. Therefore, we investigated here the potential interaction of quercetin and ferulic acid with the active sites of estrogens receptors.

#### 4.6.1. Preparation of Ligands

The compounds quercetin and ferulic acid were obtained from PUBCHEM in SDF format. The LigPrep tool in the Schrödinger Software program (version 11.5) with the OPLS3 force field was used to prepare the ligands for docking calculations. A total of 32 stereoisomers were generated for each ligand after selecting the ionization states at pH 7.0 ± 2.0 [66].

#### 4.6.2. Preparation of Proteins 

The crystal structures of estrogen alpha and estrogen beta were obtained from the protein data bank with PDB IDs 1X7B and 1U3Q, respectively. The Protein Preparation Wizard in Schrödinger-Maestro (v11.5) program was used to further refine and prepare the structures. All water molecules were removed, hydrogens were bonded to heavy atoms, methionines replaced selenomethionines, and charges and bond orders were subsequently assigned. The OPLS3 force field was utilized to carry out minimization, resulting in a maximum heavy atom RMSD of 0.30 Å. [67].

#### 4.6.3. Generation of Receptor Grid

An atom from the ligand was chosen, and a default grid box was made. The grid box had volumetric spacing of 20 × 20 × 20. Using the ‘Extra Precision’ (XP) method, the ligand was linked to the grid box produced by the protein. The XP GScore was used for assessing the results [66].

#### 4.6.4. Glide Standard Precision (SP) Ligand Docking

Flexible ligand docking using the Glide module in Schrödinger-Maestro (v11.5) was performed with standard precision (SP). Penalties were given for non-cis/trans amide bonds and the parameters for van der Waals scaling factor and partial charge cutoff of ligand atoms were both established as 0.80 and 0.15, respectively. The assessment of the final scoring was performed on energy-minimized poses, with the utilization of the Glide score to evaluate the outcomes. The top-docked pose with the lowest Glide score value was identified and recorded for each ligand [66].

### 4.7. Statistical Analysis

The data were analyzed using Graph-Pad Prism 5 software, and the Tukey’s test was employed to compare the different treatments. A significance level of *p* < 0.05 was established, and the results were presented as the mean ± standard error of the mean (s.e.m.).

## 5. Conclusions and Perspectives

This study offers scientific evidence supporting the potential benefits of quercetin and ferulic acid in addressing female infertility. It highlights their role as potentially safe anti-infertility agents when administered orally to female rats. Remarkably, our findings provide novel insights into the estrogenic properties of quercetin. Hence, conducting comprehensive investigations using cellular and animal models, along with clinical studies, would be crucial in order to elucidate the underlying mechanisms by which quercetin affects biomarkers related to fertility. Moreover, administering higher concentrations would reveal more information about the extent of the compound’s efficiency and safety. Although phytocompounds are credited with numerous health advantages, such as decreasing the chances of developing osteoporosis, heart disease, breast cancer, and menopausal symptoms, phytoestrogens could also induce endocrine disruptions, which implies they may have negative consequences on health. Therefore, special focus should be given to evidence supporting and contradicting the alleged advantages and drawbacks of phytoestrogens. Although this in silico study showed the promising therapeutic potential of quercetin against estrogen receptors, it is suitable to consider other ligands and receptor targets (e.g., luteinizing hormone (LH) and follicle-stimulating hormone (FSH)). It is also worth studying the effects of quercetin on the secretion of FSH, LH, and progesterone and thus modulating hormone imbalances and supporting reproductive health. Ultimately, this study highlights the potential therapeutic value of quercetin in addressing female infertility conditions.

## Figures and Tables

**Table 1:** Docking results with quercetin and ferulic acid in the active sites of estrogen alpha and estrogen beta receptors.

	Estrogen Alpha (PDB: 1X7B)			Estrogen Beta (PDB: 1U3Q)		
	Glide Gscore	Glide Emodel	Glide Energy	Glide Gscore	Glide Emodel	Glide Energy
Quercetin	−10.444	−82.345	−54.171	−10.662	−74.309	−51.529
Ferulic acid	−6.377	−39.698	−27.718	−6.3	−38.943	−27.308

## Data Availability

The data used to support the findings of this study are available from the corresponding author upon request.

## Sample Availability

Samples of the compounds are available from the authors.
